# In Vitro Comparative Accuracy of PSP Digital Radiography and CBCT for Detection of Broken Nickel-titanium Files in Endodontically Treated Root Canals

**DOI:** 10.31661/gmj.v13iSP1.3711

**Published:** 2024-12-29

**Authors:** Sanaz Sharifishoshtari, Mansour Jafarzadeh, Fateme Lalegani, Nima Hesabi

**Affiliations:** ^1^ Department of Oral and Maxillofacial Radiology, School of Dentistry, Ahvaz Jundishapur University of Medical Sciences, Ahvaz, Iran; ^2^ Department of Endodontics, School of Dentistry, Ahvaz Jundishapur University of Medical Sciences, Ahvaz, Iran

**Keywords:** Root Canal Therapy, Cone-beam Computed Tomography, Radiography, Dental, Digital, Nickel-titanium

## Abstract

Background: This study aimed to compare the accuracy of indirect photostimulable phosphor (PSP) digital radiography and cone-beam computed tomography (CBCT) for detection of broken nickel-titanium (NiTi) files in endodontically treated root canals. Materials and Methods: This in vitro study was conducted on 108 extracted single-rooted mandibular premolars in 4 group (n=27) of positive control (root canal instrumentation and obturation), negative control (root canal instrumentation without obturation) and two experimental groups of file fracture with and without root canal obturation. The teeth underwent PSP digital radiography and CBCT, and the radiographs were evaluated by one oral radiologist and one endodontist twice. Presence/absence of a broken file in the root canals was reported using a 4-point scale. The sensitivity, specificity, and accuracy were calculated and compared for CBCT and PSP digital radiography.Results: The sensitivity, specificity, and accuracy for detection of broken NiTi files in obturated canals were 51.9%, 59.3% and 55.6%, respectively for CBCT and 70.3%, 85%, and 77.8%, respectively for PSP radiography. These values were 81.4%, 59.3%, and 79.6%, respectively for CBCT and 85.1%, 81.4%, and 83.3%, respectively for PSP radiography in unfilled canals. PSP digital radiography was significantly superior to CBCT for detection of broken files in obturated (P=0.01) but not in unfilled (P=0.420) root canals. Conclusion: Considering the lower radiation dose and higher accuracy of PSP digital radiography than CBCT for detection of broken NiTi files in filled canals, and their comparable accuracy in unfilled canals, PSP digital radiography is recommended for this purpose.

## Introduction

Root canal complexities may be encountered during endodontic treatment and increase the risk of procedural errors and treatment failure [1]. Instrument fracture is a common procedural error that may occur due to complex morphology of the root canal system, or inappropriate use or fatigue of endodontic instruments [2][3]. Instrument fracture can prevent effective removal of the infected tissue [4] and complicate efficient root canal instrumentation [5]. Instrument fracture in the root canal also complicates access to the apical region.

Preparing curved and narrow root canals is challenging for even experienced dentists, but nickel-titanium (NiTi) files have made it easier. However, these files can break in the canal, making it hard to detect and locate them due to their low visibility on X-rays, which can complicate further treatment [3][6][7]. Early detection of a broken instrument can enhance treatment planning and prevent subsequent complications and legal problems [8]. According to Bahcall [9], separated rotary nickel-titanium (NiTi) files can pose an increased risk of post-endodontic complications, leading to extreme stress for the clinician and anxiety for the patient. Parashos and Messer also highlighted the consequences of rotary NiTi instrument fracture, emphasizing the need for prevention strategies and effective removal techniques [10]. Algarni [11] reported a high incidence of fracture in new reciprocating NiTi files, which can compromise patient outcomes if not addressed promptly.

Radiography plays a pivotal role in endodontic diagnosis, treatment planning, treatment procedure, and the outcome success assessment [12]. Radiography is required in all steps of endodontic treatment including diagnosis, working length determination, root canal preparation and obturation, and follow-up assessments [13].

Digital radiography, advanced by improvements in image acquisition and network computing, has transformed medical and dental diagnoses. It eliminates the need for processing chemicals, reduces hazardous waste, and allows for easy and quality-preserving electronic transfer of images. Digital images can also be enhanced, measured, and corrected, unlike conventional film-based radiographs [14]. Although periapical radiography provides valuable information for detection of broken instruments and their location in mesiodistal direction, it cannot provide sufficient information in buccolingual dimension [15][16]. Periapical radiography provides two-dimensional (2D) images of three-dimensional (3D) structures. Thus, overlapping and superimposition of anatomical structures often occur, resulting in some diagnostic data loss [17]. The 3D technology is suitable to overcome the limitations of 2D periapical radiography [18]. Cone-beam computed tomography (CBCT) was introduced in response to the need for a 3D imaging modality for the maxillofacial region [19]. CBCT enables recording of tooth position and related structures in different planes without superimposition of the structures. Thus, CBCT is recommended for detection of complex endodontic cases where 2D modalities fall short [20]. CBCT has become a valuable dental imaging modality due to its numerous advantages such as high accuracy, easy image acquisition, faster scanning time, and lower patient radiation dose than computed tomography [21]. In one study, the sensitivity of CBCT for detecting fractured files in root canals with filling material was lower compared to digital periapical radiography. Specifically, the sensitivity of CBCT for detecting a fractured file #10 in the canal was 63.3%. and for detecting a fractured file #35 in the canal was 36.7% [22]. Another study found that the accuracy of CBCT for detecting fractured endodontic instruments in root canals with filling material was low, and the decision to perform a CBCT examination should take this into account [23]. Despite advances in endodontic treatment, procedural errors, such as instrument fracture, continue to pose significant challenges. The detection of broken NiTi files in root canals remains a daunting task, particularly in complex cases. Traditional radiographic methods, including periapical radiography, have limitations in providing accurate diagnoses, particularly in the buccolingual dimension. The introduction of CBCT has addressed some of these limitations, but its accuracy in detecting broken NiTi files in root canals with filling material has been questioned. Furthermore, the radiation dose associated with CBCT is a concern. In light of these limitations, this study aimed to investigate the accuracy of indirect PSP digital radiography and CBCT in detecting broken NiTi files in endodontically treated root canals, with a focus on comparing their diagnostic efficacy and radiation doses.

## Materials and Methods

**Figure-1:**
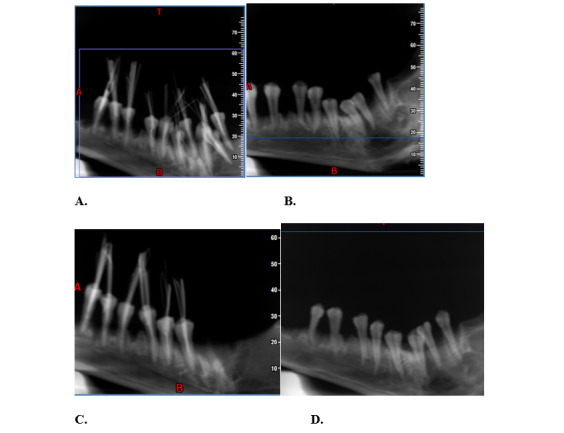
Figure 1. CBCT images of the teeth; (A) presence of broken file + obturation, (B) presence of broken file, no obturation. (C) absence of broken file + obturation, (D) absence of broken file, no obturation

**Figure-2:**
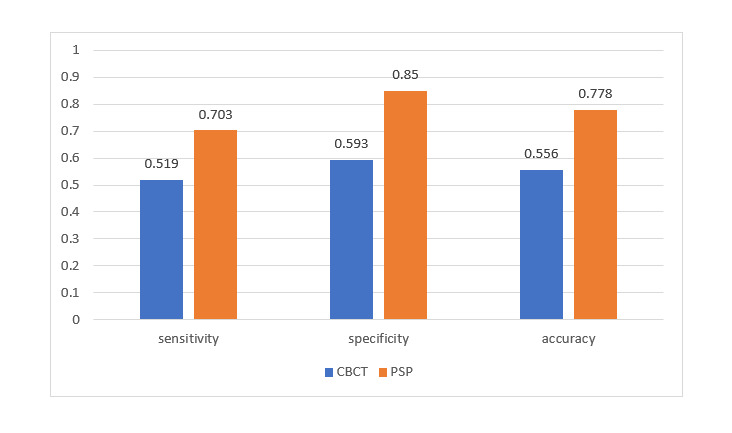
Figure 2. Sensitivity, specificity and accuracy of CBCT and PSP digital radiography in detection of broken NiTi files in root canals filled with gutta-percha

This in vitro, experimental study was conducted on 108 single-rooted mandibular premolars extracted for orthodontic or periodontal reasons. The study protocol was approved by the ethics committee of Ahwaz Jundishapur University of Medical Sciences. (IR.AJUMS.REC.1400.543)

Sample Size

The sample size was calculated to be 108 assuming alpha=0.05, beta=0.2, and 95% confidence interval, using the sample size calculation formula.

Eligibility Criteria

Single-rooted mandibular premolars with no fracture, crack, and internal/external root resorption were selected.

Specimen Preparation

The teeth were randomly assigned to four groups (n=27). To ensure an unbiased and equitable distribution, a random number generator was used to allocate each tooth to one of the four groups. Standard access cavities were created in teeth using a medium-size round bur and high-speed hand-piece. A #15 K-file was introduced into the canal until its tip passed the apex by 1 mm; 1 mm was subtracted from this length to determine the working length. The root canals were then instrumented by RaCe rotary system (FKG, Switzerland) up to #30/6% as instructed by the manufacturer.

In group 1, the root canals were instrumented as explained above and were obturated with gutta-percha and AH26 sealer (Dentsply Sirona, Switzerland). This group served as the positive control group and did not have any broken file in the canals.

In group 2, the apical third of the canals was instrumented with old defective files in order for the file to fracture in the canal. After file fracture, the root canals were obturated with gutta-percha and AH26 sealer.

In group 3, the teeth were instrumented without any file fracture in the canal but were not obturated. This group served as the negative control.

In group 4, the apical third of the canals was instrumented with old defective files in order for the file to fracture in the canal. The root canals were not obturated after file fracture.

To simulate the periodontal ligament, the roots were coated with one layer of thin wax and mounted in dry sheep mandible.

Radiography

The teeth then underwent CBCT using NewTom VGi CBCT scanner (Italy) with 110 kVp, 45.93 mAs, 5.4 s exposure time, and 8 x 8 Cm field of view, in high-resolution mode. then the images were transferred to NNT software (Newtom,Italy) for processing, and saved.

Next, the teeth underwent digital PSP radiography (de Gotzen, Italy) with the exposure settings of 70 kVp, 8 mA, and 0.32 s time using a PSP plate. Indirect PSP digital periapical radiographs were obtained from the mounted teeth by the parallel technique and processed by Digora Optime (Soredex, Finland). The PSP plate was held by a holder at 10 cm distance from the X-ray tube and the mounted teeth were positioned at equal distance from both the tube and the sensor. The images were transferred to Scanora software (Soredex,Italy) and saved with no image manipulation or enhancement by the digital filters. All CBCT scans and digital radiographs (Figures-1 and -2) were arranged in a PowerPoint slide show and viewed by an oral radiologist and an endodontist blinded to the group allocation of the teeth. The two observers were requested to view the images twice with a 2-week interval under similar environmental and lighting conditions. They were asked to express their expert opinion regarding presence/absence of a broken file in the canals using a 5-point scale as follows: 0: Definitely absent, 1: probably absent, 2: indefinite, 3: probably present, 4: definitely present. The sensitivity, specificity, and accuracy were calculated and compared for CBCT and PSP digital radiography.

Prior to the study, both the CBCT and PSP radiography equipment underwent calibration procedures according to the manufacturers’ guidelines to ensure optimal performance. Quality checks on the images were performed to ensure consistency and clarity, and any images that did not meet the predefined quality standards were excluded from the analysis.

For both imaging modalities, the settings were kept consistent across all specimens. The CBCT scans were performed using the same settings of 110 kVp, 45.93 mAs, 5.4 s exposure time, and 8 x 8 Cm field of view in high-resolution mode. The PSP digital radiographs were obtained with consistent exposure settings of 70 kVp, 8 mA, and 0.32 s exposure time. The specimens were positioned in the same way for each imaging modality to ensure standardized conditions. Specifically, the PSP plate was held by a holder at a 10 cm distance from the X-ray tube, and the mounted teeth were positioned at an equal distance from both the tube and the sensor.

Before the study, the two observers (an oral radiologist and an endodontist) were trained on the use of the 5-point scale to ensure a common understanding of the criteria.

Statistical Analysis

Data were analyzed using SPSS version 26 (SPSS Inc., IL, USA) by the Chi-square test and receiver operating characteristic (ROC) curve. P<0.05 was considered statistically significant.

## Results

**Figure-3:**
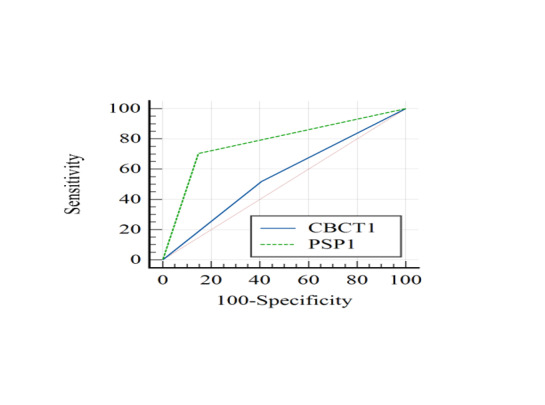
Figure 3. ROC curve for the comparison of PSP digital radiography and CBCT for detection of broken NiTi files in root canals filled with gutta-percha

**Figure-4:**
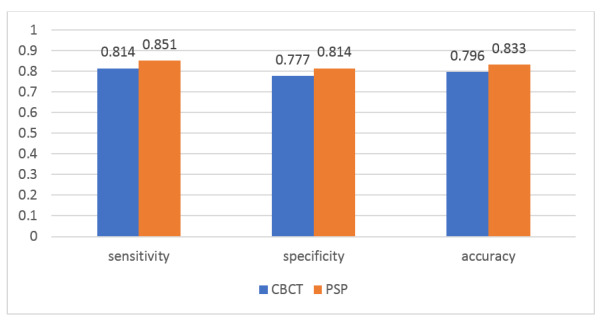
Figure 4. Sensitivity, specificity and accuracy of CBCT and PSP digital radiography for detection of broken NiTi files in empty root canals

**Figure-5:**
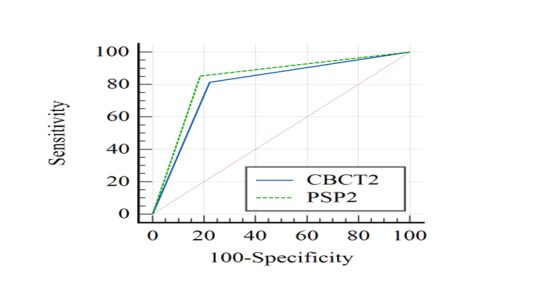
Figure 5. ROC curve for the comparison of PSP digital radiography and CBCT for detection of broken NiTi file in empty root canals

**Table:** Table1. Sensitivity, Specificity, and Accuracy of CBCT for Detection of Broken NiTi Files

**Group**	**Diagnosis**		**Number of teeth**		**Sensitivity**	**Specificity**	**Accuracy**
		**Absence of broken file**	**Presence of broken file**	**Total**			
	Presence of broken file	11	14	25			
**Obturated canals**	Absence of broken file	16	13	29	0.519	0.593	0.556
	Total	27	27	54			
	Presence of broken file	6	22	28			
**Unfilled canals**	Absence of broken file	21	5	27	0.814	0.777	0.796
	Total	27	27	54			

Sensitivity, Specificity, and Accuracy of CBCT for Detection of Broken NiTi Files in Obturated and Unfilled Root Canals

As shown in Table-1, the accuracy of CBCT was 55.6% for detection of broken NiTi files in root canals filled with gutta-percha and 79.6% for detection of broken NiTi files in unfilled root canals. The accuracy of CBCT for detection of broken NiTi files in unfilled root canals was significantly higher than its accuracy in obturated canals (P=0.004).

Sensitivity, Specificity, and Accuracy of PSP Digital Radiography for Detection of Broken NiTi Files in Obturated and Unfilled Root Canals

The accuracy of PSP digital radiography was 77.8% for detection of broken NiTi files in obturated root canals and 83.3% for detection of broken NiTi files in unfilled root canals. No significant difference was found in the accuracy of PSP digital radiography for detection of broken NiTi files in obturated and unfilled root canals (P=0.492, Table-2).

Comparison of Sensitivity, Specificity and Accuracy of CBCT and PSP Digital Radiography for Detection of Broken NiTi Files in Obturated Root Canals

PSP digital radiography had a significantly higher accuracy than CBCT for detection of broken files in root canals filled with gutta percha (P=0.01, Figures-2 and -3). The sensitivity (P<0.05) and specificity (P<0.05) of PSP digital radiography were both significantly higher than CBCT in detection of broken files in obturated root canals.

Comparison of Sensitivity, Specificity and Accuracy of CBCT and PSP Digital Radiography for Detection of Broken NiTi Files in Unfilled Root Canals

No significant difference was found in the accuracy of CBCT and PSP digital radiography for detection of broken NiTi files in unfilled root canals (P=0.420, Figures-4 and -5). The difference in sensitivity (P>0.05) and specificity (P>0.05) of CBCT and PSP digital radiography was not significant either.

## Discussion

**Table:** Table2. Sensitivity, Specificity, and Accuracy of PSP Digital Radiography for Detection of Broken NiTi Files

**Group**	**Diagnosis**		**Number of teeth**		**Sensitivity**	**Specificity**	**Accuracy**
		**Absence of broken file**	**Presence of broken file**	**Total**			
	Presence of broken file	4	19	28			
**Obturated canals**	Absence of broken file	23	8	26	0.703	0.85	0.778
	Total	27	27	54			
	Presence of broken file	5	23	28			
**Unfilled canals**	Absence of broken file	22	4	26	0.851	0.814	0.833
	Total	27	27	54			

This study compared the accuracy of PSP digital radiography and CBCT for detection of broken NiTi files in endodontically treated root canals. The results showed significantly higher accuracy of PSP digital radiography in detection of broken NiTi files in root canals filled with gutta-percha. However, the difference between the two modalities was not significant in absence of gutta-percha.

Madian et al. [24] compared the diagnostic accuracy of digital periapical radiography, and CBCT with/without metal artifact reduction algorithm for detection of broken endodontic files. They found no significant difference in diagnostic accuracy of the three modalities in curved unfilled canals. However, in straight obturated canals, the accuracy of periapical radiography was significantly higher than that of CBCT with/without metal artifact reduction algorithm. Their results were in agreement with the present findings although they used the direct digital technique.

Alemam et al. [25] compared the accuracy, sensitivity, and specificity of CBCT, conventional radiography, and semi-direct digital radiography for detection of broken instruments in the canal. They found no significant difference in sensitivity, specificity, or accuracy of the three modalities in unfilled canals. In filled canals, however, CBCT showed lower accuracy and sensitivity than digital and conventional periapical radiography, with no significant difference between the latter two. Their results were in line with the present findings. Ayatollahi et al. [22] compared the diagnostic accuracy of CBCT and indirect digital periapical radiography for detection of broken file segments with two different lengths, and showed that digital radiography had superior sensitivity, specificity, accuracy, and positive and negative predictive values than CBCT for both lengths of the broken segments, which was in accordance with the present results. Brito et al. [23] compared indirect digital radiography with different angulations and CBCT. They reported that in empty root canals, all modalities had high accuracy with no significant difference. However, in obturated canals, CBCT had lower accuracy. They recommended direct digital radiography for detection of broken instruments and strip perforations in the canals. Despite different techniques, their results were generally in line with the present findings. Abdinian et al. [26] compared CBCT and indirect digital radiography with two different horizontal angulations for detection of broken files. They found that CBCT had higher accuracy in detection of strip perforation while PSP digital radiography had higher accuracy for detection of broken files. Despite a different methodology, their results were similar to the present findings.

D’Addazio et al. [20] compared CBCT and conventional periapical radiography for detection of simulated endodontic complexities and file fracture. They showed that CBCT was superior only for detection of external root resorption defects while periapical radiography was superior for detection of broken endodontic files, root perforations, and deviated cast posts. Despite different methodology, their results were in agreement with the present findings. Haghanifar et al. [27] compared CBCT and digital periapical radiography with three different angulations for detection of root perforation in obturated and unfilled mandibular molars. They concluded that digital radiography was more reliable than CBCT for detection of perforation in obturated canals. However, CBCT was superior in unfilled canals. Similar to the present study, they indicated that root filling can significantly decrease the diagnostic quality of CBCT, although they evaluated root perforation and not file fracture.

CBCT artifacts are a main reason for lower diagnostic accuracy of CBCT than PSP digital radiography for detection of broken files in root canals [28][29][30][31][32]. Metal posts and non-metal root filling materials can all cause CBCT artifacts. Similar radiopacity of NiTi files and gutta-percha further complicates their differentiation [33], and can cause significant CBCT artifacts especially when a high-density object is present in the scanned volume [31]. Moreover, metal posts in the canal cause artifacts that decrease image resolution and increase the frequency of false positive results [20]. On the other hand, presence of gutta-percha in the canal may decrease the sensitivity and specificity of CBCT. Type of root filling material and it relative radiopacity as well as CBCT parameters such as voxel size, and field of view may also affect artifact generation and intensity. The artifacts caused by root filling materials on CBCT scans are much higher than those on periapical radiographs, and significantly decrease the diagnostic efficacy of CBCT [34].

This study had some limitations. Only one brand of CBCT scanner and one field of view were evaluated in this study, and enhancement filters were not used for PSP digital radiographs. Future studies are required on other brands of CBCT scanners with smaller fields of view, and also PSP enhancement filters. Furthermore, the diagnostic accuracy of CBCT and PSP digital radiography may be compared for detection of other types of broken instruments in the canals such as Gates-Glidden drills, peeso reamers, Lentulo spiral, and different types of endodontic files.

## Conclusion

The study’s findings support the recommendation of PSP digital radiography for detecting broken NiTi files, especially in obturated canals, due to its higher accuracy and lower radiation dose. In unfilled canals, both techniques are comparable, offering clinicians the flexibility to choose based on availability and patient needs. This research provides valuable insights for endodontic practitioners, enhancing the precision and safety of their diagnostic processes.

## Conflict of Interest

The authors have no competing interests to declare that are relevant to the content of this article.
